# Poly-γ-Glutamic Acid: Biodegradable Polymer for Potential Protection of Beneficial Viruses

**DOI:** 10.3390/ma9010028

**Published:** 2016-01-06

**Authors:** Ibrahim R. Khalil, Victor U. Irorere, Iza Radecka, Alan T. H. Burns, Marek Kowalczuk, Jessica L. Mason, Martin P. Khechara

**Affiliations:** 1 Faculty of Science and Engineering, University of Wolverhampton, Wulfruna Street, Wolverhampton WV1 1LY, UK; Irorere-V@email.ulster.ac.uk (V.U.I.); I.Radecka@wlv.ac.uk (I.R.); A.T.Burns@wlv.ac.uk (A.T.H.B.); M.Kowalczuk@wlv.ac.uk (M.K.); J.Mason@wlv.ac.uk (J.L.M.); 2 Department of Biology, College of Science, Tikrit University, Tikrit PO Box 42, Iraq; 3 Center of Polymer and Carbon Materials, Polish Academy of Sciences, ul. M. Curie-Skłodowskiej 34, Zabrze 41-819, Poland

**Keywords:** biodegradable polymer, γ-PGA, bacteriophage

## Abstract

Poly-γ-glutamic acid (γ-PGA) is a naturally occurring polymer, which due to its biodegradable, non-toxic and non-immunogenic properties has been used successfully in the food, medical and wastewater industries. A major hurdle in bacteriophage application is the inability of phage to persist for extended periods in the environment due to their susceptibility to environmental factors such as temperature, sunlight, desiccation and irradiation. Thus, the aim of this study was to protect useful phage from the harmful effect of these environmental factors using the γ-PGA biodegradable polymer. In addition, the association between γ-PGA and phage was investigated. Formulated phage (with 1% γ-PGA) and non-formulated phage were exposed to 50 °C. A clear difference was noticed as viability of non-formulated phage was reduced to 21% at log_10_ 1.3 PFU/mL, while phage formulated with γ-PGA was 84% at log_10_ 5.2 PFU/mL after 24 h of exposure. In addition, formulated phage remained viable at log_10_ 2.5 PFU/mL even after 24 h of exposure at pH 3 solution. In contrast, non-formulated phages were totally inactivated after the same time of exposure. In addition, non-formulated phages when exposed to UV irradiation died within 10 min. In contrast also phages formulated with 1% γ-PGA had a viability of log_10_ 4.1 PFU/mL at the same exposure time. Microscopy showed a clear interaction between γ-PGA and phages. In conclusion, the results suggest that γ-PGA has an unique protective effect on phage particles.

## 1. Introduction

Poly-γ-glutamic acid is a naturally occurring polymer synthesized by various microbial strains especially Bacillus species [1,2]. It is a homopolyamide, composed of glutamic acid monomers connected by amide linkages between α-amino and γ-carboxyl groups. The polymer γ-PGA can be obtained in either L or D or both L and D isomeric forms depending on the bacterial strain or on the productive media employed in its production. This polymer can be water insoluble (free acid form) or completely water soluble as its salt form with a variety of cations (Na^+^, Mg^2+^, K^+^, NH^4+^ or Ca^2+^) [3,4,5]. γ-PGA has been used widely in drug delivery platforms, because it has carboxyl groups on the side chains, this group offers attachment points for the conjugation of chemotherapeutic agents; thus making the drug more soluble and easy for controlled release. It has also been used for protein and vaccine encapsulation, immobilization or adsorption for delivery to specific action sites [6,7,8]. Although no report of viral γ-PGA interaction was found in the literature, the encapsulation or immobilization of vaccines with this biodegradable polymers and its use in the protection of probiotic bacteria from harsh environmental conditions [7,8,9,10] suggest that γ-PGA can be used as an immobilization or encapsulating agent for bacteriophage to offer protection from harmful environmental conditions.

Bacteriophages (phages) are viruses that invade specific bacteria. Virulent or lytic phages infect and kill their hosts. Their high specificity and rapid killing kinetics against their target species plus their safety profile make them particularly attractive antibacterial natural products [11,12]. Antibiotic resistance of bacterial pathogens is presently a source of major concern to modern medicine and its incidence has grown sharply in recent years [13,14]. As a result, improving phage therapy may represent an important step in the fight against these resistant bacteria. There are many advantages in the use of phage for biological control. For instance, the specificity of some phages to a single bacterial species, or even strain allows safe targeting to the pathogenic bacteria without causing any harm to the normal flora when used for therapeutic purposes. Secondly, their low cost of production and ability to proliferate once inside their target cell makes them suitable antibacterial agents with regards to both economic cost and dosage [15,16,17,18]. Furthermore, they have a high success rate against the target organism with no reports of harmful side effects to humans to date [19,20].

However, the ability of phage to persist for extended periods is limited by many factors, particularly environmental factors such as sunlight, irradiation (UV irradiation), temperature, desiccation, and exposure to copper bactericides [21,22,23,24]. High temperature directly affects the amino acids of bacteriophage, by fracturing their bonds and denaturing the proteins of the phage particle [20,21] while UV radiation results in direct DNA damage by causing lesions in cellular DNA that consequently obstruct RNA transcription and DNA replication [24]. It is, therefore, necessary that methods be developed to protect phages from these environmental factors in order to improve their persistence in the environment for efficient storage and potential use as topical or environmental antibacterial agents. Previously, we have shown that the biodegradable polymer poly-γ-glutamic acid (γ-PGA) can be used to protect beneficial bacteria from various environmental factors during their various stages of preparation, storage and ingestion [9,10]. We therefore hypothesized that this polymer could also be applied in bacteriophage protection from harmful environmental factors. Thus, this research was aimed at investigating the survival of bacteriophage under different environmental conditions when protected with γ-PGA.

## 2. Results

### 2.1. Identification of γ-PGA

**Figure 1:**
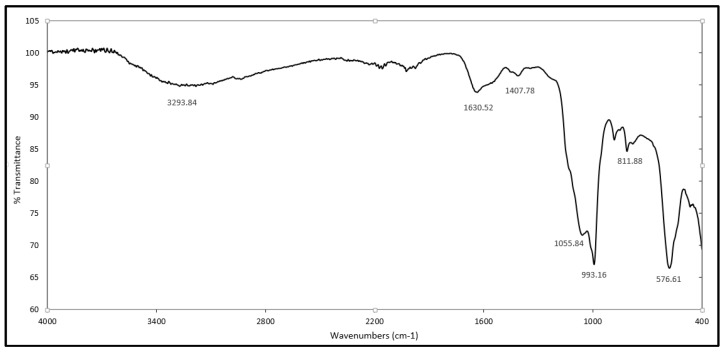
FT-IR absorption spectrum of γ-PGA produced by *B. subtilis* natto in G.S medium. The spectrum is the mean of 3 spectra.

FT-IR and ^1^H-NMR spectroscopies were used in the identification of the γ-PGA produced. The spectrum shown is a mean of 3 spectra. The IR spectra (Figure 1) gave peaks specific to γ-PGA [1] with the strong amide absorption seen at wavenumbers of 1620.52 (cm^−1^). The peaks at 1407.78 (cm^−1^) and 1055.84 (cm^−1^) are representative of C=O and C-N groups respectively while the stretch seen at ~3293 (cm^−1^) is representative of the hydroxyl group.

**Figure 2:**
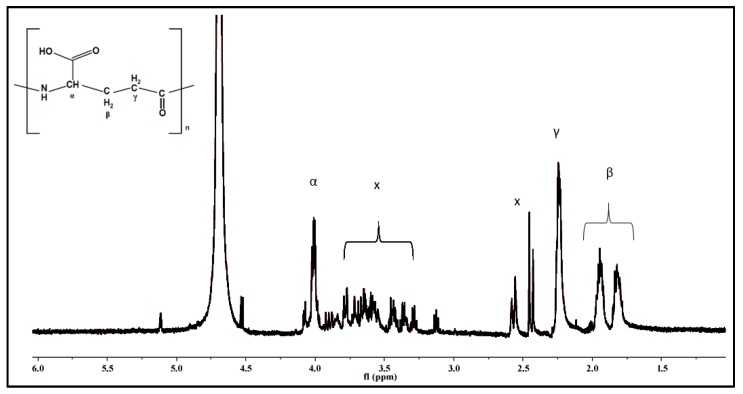
^1^H-NMR spectrum of γ-PGA. The peak 4.7 ppm corresponds to the D_2_O used as a solvent. X-marked peaks indicate impurity peaks, most of which are amino acids residues.

Also the ^1^H-NMR spectrum (Figure 2) confirms the sole presence of γ-PGA in the synthesized samples. X indicates impurity peaks and the extraordinary high peak is the Deuterium Oxide (D_2_O) used as a solvent. The number average molecular mass (M_n_) analyzed by GPC was 482,000 and dispersity was 1.3.

### 2.2. Effect of Temperature on Formulated and Non-Formulated Phage

**Figure 3:**
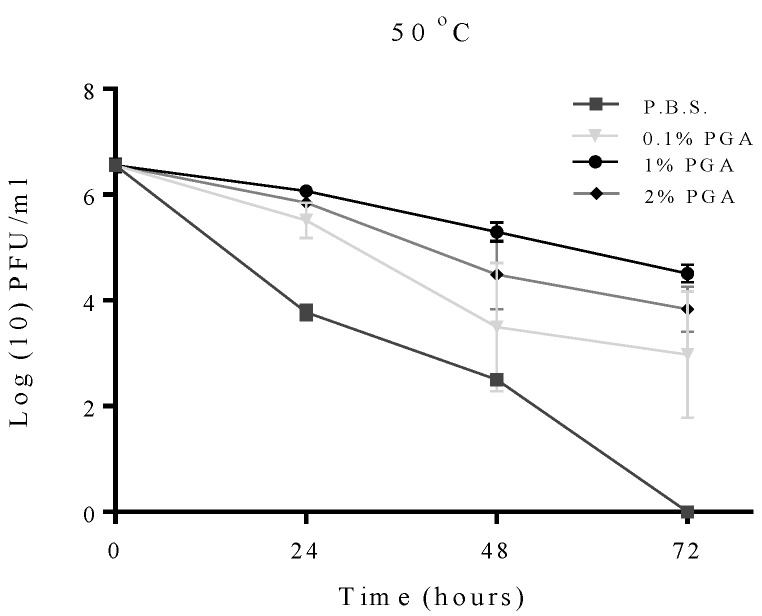
Effect of different concentrations of γ-PGA on MS2 viability at 50 °C, over the time course of 72 h. Experiments were conducted in quadruplicate (n = 4). Error bars represent the standard error of the mean. (PBS = non-formulated phage in phosphate buffered saline, used as a control; PGA = formulated phage with poly-gamma-glutamic acid).

The survival rate of formulated and non-formulated phage was evaluated via a series of time course experiments at different temperatures. The effect of γ-PGA concentration on the viability of formulated phage was initially studied at 50 °C. The results obtained show an increase in viability at all incubation time with increasing concentration of γ-PGA (Figure 3). The difference in viability between 1% and 2% is however significant but with little biological relevance (P ≤ 0.05). Thus, 1% γ-PGA formulation was used in subsequent experiment.

**Figure 4:**
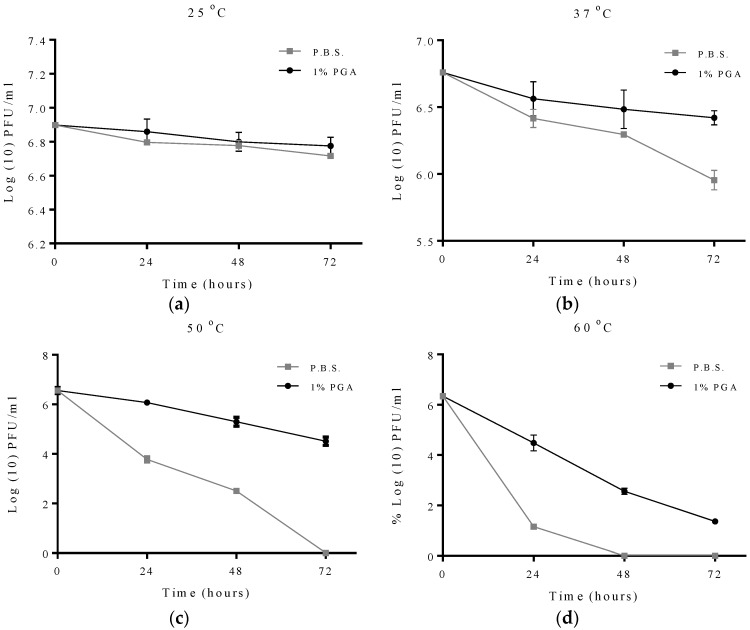
Protective effect of γ-PGA on MS2 phage at different temperatures. Data points were measured at 24, 48 and 72 h. All experiments were conducted in quadruplicate (n = 4). Error bars represent the standard error of the mean. Formulated and non-formulated MS2 phage exposed to (**a**) 25 °C; (**b**) 37 °C; (**c**) 50 °C; (**d**) 60 °C.

For the effect of increasing temperature on MS2 viability, viability of formulated and non-formulated phage at different temperatures was assessed at 24, 48 and 72 h. The results obtained showed that at 25 °C there was no significant change in MS2 viability under either condition (Figure 4). At 37 °C, no significant difference was observed in the viability of formulated and non-formulated phage at 24 h and 8 h while at 72 h the viability of formulated phage (Log_10_ 6.4 PFU/mL) was significantly higher than non-formulated phage (Log_10_ 5.9 PFU/mL).

When the incubation temperature was increased to 50 °C, significant differences can be observed in the viability of formulated and non-formulated phages from 24 h to 72 h of incubation, with a complete loss in viability observed at 72 h for non-formulated phage while formulated phage had a viability of Log_10_ 5.2 PFU/mL. Increasing the incubation temperature further to 60 °C reveals a similar trend to an incubation temperature of 50 °C in the differences in viability between formulated and non-formulated phage over a 72 h period. However, complete loss in viability was observed at 48 h for non-formulated phage while formulated phage had a viability of Log_10_ 1.3 PFU/mL even after 72 h of incubation (Figure 4).

**Figure 5:**
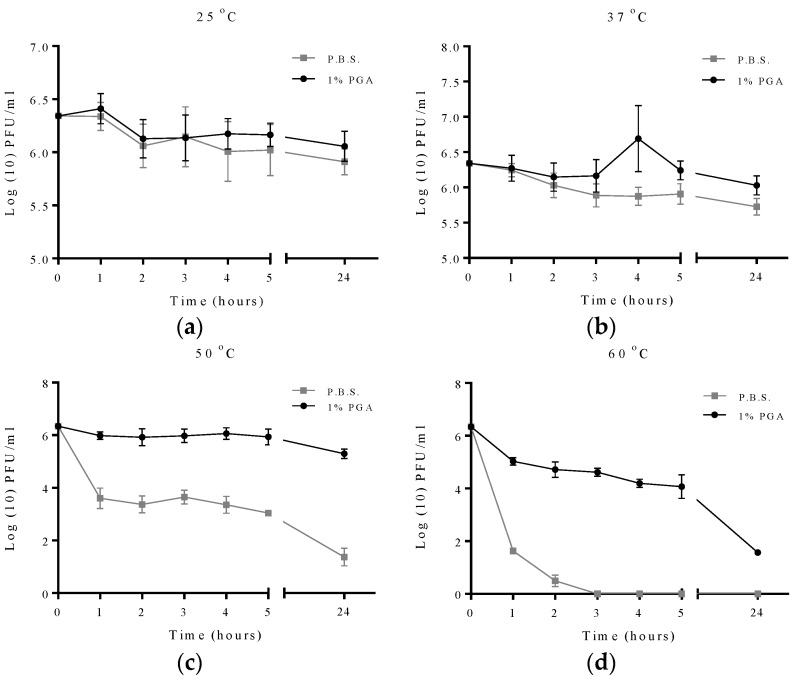
Effect of elevated temperature on formulated and non-formulated T2 phage longevity. Experiments were measured at 1, 2, 3, 4, 5 and 24 h. All experiments were conducted in quadruplicate (n = 4). Error bars represent the standard error of the mean. Formulated and non-formulated T2 phage exposed to (**a**) 25 °C; (**b**) 37 °C; (**c**) 50 °C; (**d**) 60 °C. However, at 50 °C a significant difference was observed in the viability between formulated and non-formulated phage at all time points with formulated phage having a viability of 5.3 Log_10_ PFU/mL after 24 h of incubation compared to non-formulated phage with a viability of 1.3 Log_10_ PFU/mL, representing an 84% difference in viability. Similar results were obtained at 60 °C as the survival rate of non-formulated phage dramatically declined (Figure 5) and completely disappeared after 3 h while formulated phage was still viable with a survival rate of 1.5 Log_10_ PFU/mL.

For T2 phage, viability of formulated and non-formulated phage was assessed at 1, 2, 3, 4, 5 and 24 h of incubation at the different temperature studied. At 25 °C there was no significance difference between formulated and non-formulated T2 phage after 24 h of incubation (Figure 5). At 37 °C the difference in viability between formulated T2 phage and non-formulated phage was significant at all time points studied.

**Figure 6:**
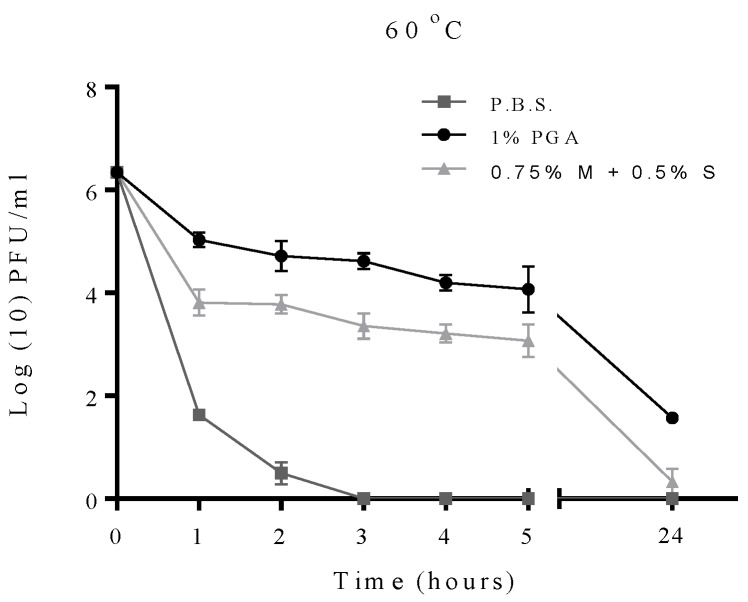
A comparison between formulated T2 phage with γ-PGA and formulated T2 phage with 0.75% of skimmed milk and 0.5% of sucrose (0.75% M + 0.5 S) after exposure to 60 °C for 24 h. Experiments were conducted in quadruplicate (n = 4). Error bars represent the standard error of the mean.

In order to investigate which formulation had the better protective effect, a comparison between T2 phage formulated with γ-PGA or with 0.75% skimmed milk and 0.5% sucrose as described previously [24] was conducted. Both formulations were exposed to 60 °C for 24 h (Figure 6). The viability of formulated phage with γ-PGA was greater than the non-γ-PGA formulation as phages formulated with γ-PGA showing 1.5 log unit of viability, while the milk formulated phage was almost inactivated after 24 h of exposure.

### 2.3. Effects of pH on Formulated and Non-Formulated Phage

**Figure 7:**
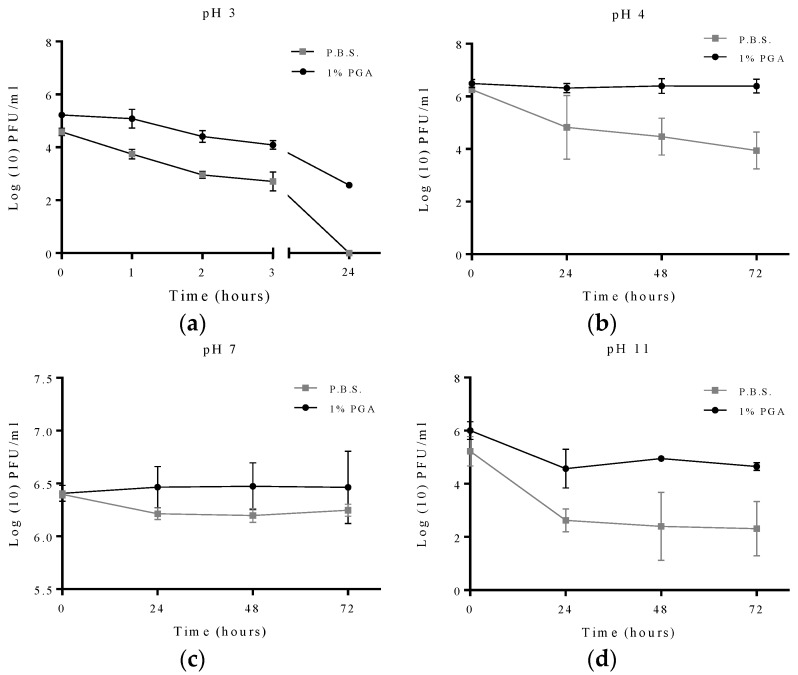
The protective effect of γ-PGA on T2 phage at different pH solutions. Experimental points measured at 0, 24, 48 and 72 h, and conducted in quadruplicate (n = 4). Readings from the pH 3 experiment were measured at 0, 1, 2, 3 and 24 h. Error bars represent the standard error of the mean. Formulated and non-formulated T2 phage exposed to solution of (**a**) pH 3; (**b**) pH 4; (**c**) pH 7; (**d**) pH 11.

The effect of pH on the viability of formulated and non-formulated T2 phage was studied using different pH solutions. Viability was determined at 0, 24, 48 and 72 h of exposure except for pH 3 where viability was determined at 0, 1, 2, 3 and 24 h of exposure as preliminary experiment showed no viability for either formulated or non-formulated phage after 48 h (results not shown). At pH 11, formulated phage survived well when compared to non-formulated phage as the formulated phage population remained at 4.6 Log_10_ PFU/mL after 72 h of exposure compared to non-formulated phage viability which was 2.3 Log_10_ PFU/mL. At pH 4, no significant reduction in viability was observed in the formulated phage throughout the 72 h period of exposure while non-formulated phage had a significant reduction in viability of approximately 2.5 Log_10_ PFU/mL (Figure 7). When the pH was reduced to 3, a complete loss in viability was observed for non-formulated phage after 24 h while formulated phage still had a viability of about 2.5 Log_10_ PFU/mL (Figure 7).

### 2.4. Effect of UV Irradiation on Formulated and Non-Formulated Phage

**Figure 8:**
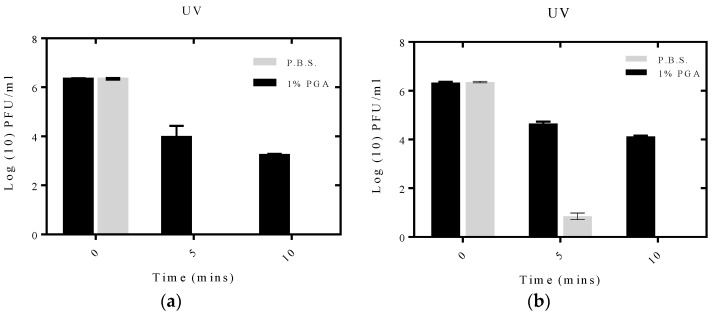
Effect of UV irradiation on formulated on non-formulated phage population: (**a**) Protective effect of γ-PGA on T2 phage; (**b**) Protective effect of γ-PGA on MS2 phage. Readings were measured at 0, 5 and 10 min, at a wave length of 245 nm. All experiments were conducted in quadruplicate (n = 4). Error bars represent the standard error of the mean.

To investigate the protective effect of γ-PGA, non-formulated or formulated phage with γ-PGA were exposed to ultraviolet light (UV) for 5 and 10 min, and viability measured with the overlay plaque assay. Formulated T2 phage showed better survival after exposure to UV irradiation compared to non-formulated phage, which showed complete loss of viability after 5 min of exposure (Figure 8a). The viability of formulated phage was above 50% at 3.2 Log_10_ PFU/mL even after 10 min of exposure to UV irradiation. 

Formulated and non-formulated MS2 phages were also exposed to UV irradiation. The results obtained showed a significant decrease in viability of non-formulated MS2 to 0.8 Log_10_ PFU/mL after 5 min and a complete loss in viability after 10 min of exposure to UV irradiation. On the other hand, formulated MS2 retained viability after 10 min of exposure with a viability of 4.1 Log_10_ PFU/mL (Figure 8b).

### 2.5. Formulated and Non-formulated Phage Microscopy

**Figure 9:**
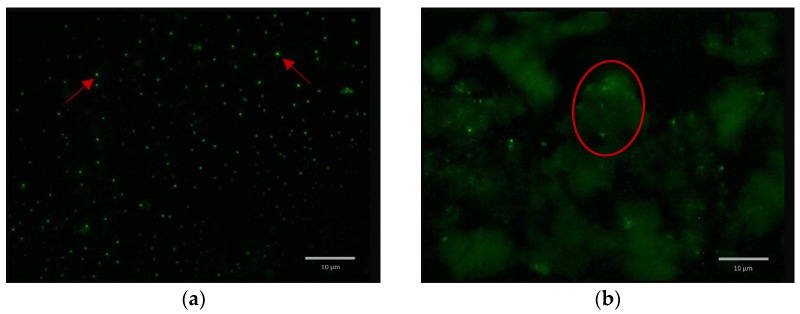
SYBR green I (2.5% v/v) stained samples were investigated under Fluorescence Microscopy: (**a**) T2 in PBS (non-formulated phage) well dispersed as represented by well-spaced bright particles as shown by red arrows; (**b**) T2 formulated with γ-PGA, clearly virion particles are associated with γ-PGA as shown within red ring. Scale bar, 10 μm.

In order to investigate the interaction between the phage and γ-PGA, T2 in PBS and formulated T2 was stained with 2.5% (v/v) SYBR green I and examined under high magnification using fluorescence microscopy. Non-formulated phage (T2 in PBS were well dispersed represented by well-spaced bright particles. In contrast, the formulated phage were associated with γ-PGA (Figure 9), as formulated phages are seen to aggregate in characteristic clumps within and around γ-PGA. 

## 3. Discussion

Poly-γ-glutamic acid (γ-PGA) is a biodegradable, biocompatible and non-toxic polymer. These characteristics make it a particularly attractive polymer for a wide range of potential applications in the biomedical and environmental industries. Their solubility in water is also a characteristic that makes it well suited for these applications compared to other polymers that are soluble only in organic solvents, some of which may have harmful environmental and biological implications. The ability of γ-PGA to protect and effectively deliver a wide range of drugs to their target site has been studied extensively [1,25,26]. In addition, the polymer has been used in the protection and delivery of genetic materials and vaccines [1]. Recently, γ-PGA was shown to effectively protect probiotic bacteria from the effect of harsh environmental conditions for effective storage and enhanced delivery to their host [9]. However, the use of these polymers to protect active phage particles from harsh environmental conditions has previously not been studied. 

Phages are considered to have possible applications as an alternative or as a complement to antibacterials for humans and animals or as chemical disinfectants on hands, plant leaves and hard surfaces [24,27]. There are many advantages of using phages as an alternative to antibiotics or for biological control. One major example is that phages are specific in their action and if used as disinfectants can help to stop the spread of a particular pathogen in the environment without interfering with normal microflora. The ability of phages to replicate rapidly [15,16,17,18] means that they do not need to be applied in high amounts as even a small number of phage particles can be highly effective if protected from harsh environmental conditions. Moreover, phages and their bacterial hosts are an attractive alternative among possible model systems as they allow facile manipulation of conditions such as: host, population parameters, transmission rates and environmental conditions [28]. Phages are not especially demanding with regard to facilities or equipment and are rapidly regenerated without any difficulty and at a relatively low cost [29]. However, the ability of phage to persist for extended periods is limited by many factors including UV irradiation, temperature, acidity and desiccation [21,23,24].

In this work, the survival rates of formulated and non-formulated phage were evaluated via a series of time course experiments under different adverse conditions. This study demonstrated that γ-PGA can efficiently protect MS2 and T2 phage in environments with temperatures as high as 60 °C. The observed data shows that γ-PGA increases survival of MS2 bacteriophage when it is exposed to increasing temperatures including 37 °C, 50 °C and 60 °C (Figure 4). A previous study using the integrated virus detection system (IVDS) found MS2 phages can be detected when temperatures are increased to 63 °C for half an hour, but longer exposure or increasing temperature results in a quick decrease in detectable MS2 particles [30]. However, we show here that MS2 phages without γ-PGA were detected even after 24 h of exposure to 60 °C but with very low viable number. When γ-PGA was present, this loss in viability was less rapid. This finding suggests that protection might occur through physical protection of the virus particle as γ-PGA mask the phage and decrease the heat levels reaching viral particles, or the high amino acid content in γ-PGA may be favourable for virion survival. The biopolymer γ-PGA is stable at all temperatures used in this study as it has a melting point as high as 206 °C. To enhance γ-PGA production, *Bacillus licheniformis* can be heated to 50 °C, which actually increases γ-PGA yield by 66% [31]. This suggests a role of γ-PGA production in the cell as a heat-shock response. As γ-PGA protects the host from heat, it also then may protect MS2 from heat in similar manner, due to its chemical and physical properties shielding the viral capsid and delaying denaturation. An interesting finding is that the protective effect of γ-PGA is not dose-dependent, practically, at 50 °C. The data suggests 1% γ-PGA may be more protective than 2% γ-PGA in case of MS2 phage. It could be that higher concentrations of γ-PGA may prevent interaction of phage virions with the bacterial host. Although at this point this is only a qualitative observation and requires further investigation.

The phages T4 and T2 are members of the *Myoviriadae* family and are stable at a pH range of 5–9, below or above this range phage survival is significantly decreased [21]. Survival of formulated T2 phage was remarkably improved at different pH values. All experiments at different pH values were evaluated at 0, 24, 48 and 72 h. the pH value of 3 was also examined at 0, 1, 2, 3 and 24 h, there was no observation of any survival rate after 48 h, from either formulated or non-formulated phage. The polymer used in our work is a pH sensitive polymer. In highly acidic environments the polymer is stable as it forms an alpha-helical structure. This conformational change which is thought to allow phage to be effectively “wrapped up” or shielded by the now, filament-like polymer from acidic environments. This effect has already been investigated by similar study; Bhat and co-workers demonstrated that formulating probiotic bacteria with γ-PGA increases the survival rate remarkably in extreme acidic environment [9]. This biopolymer is able to form different engineering structures at different pH. Ho *et al.* (2006) observed that γ-PGA forms a linear random-coil formation at neutral pH [32]. For that reason, the protective effect of pH 11 was not fully understood. It might be the linear random-coil formation physically masks the phage and shields it from the harsh surrounding environment. It was also observed that with longer duration of the pH 11 experiment the pH level decreased, especially on the third day of exposure (data not shown). However, further investigations as to the possible buffering effect of γ-PGA need to be performed. 

The difference in survival rates between formulated and non-formulated T2 phage was significant (P < 0.05) at all pH values tested. However, there were no statistically significant results in the reduction of MS2 phage viability with or without γ-PGA formulation (results not shown). Phage MS2 is stable over a wide range of pH values. Previous work has found MS2 is stable under strong acidic conditions as low as pH 1.4 [30]. This may explain why we did not observe a difference in formulated and non-formulated MS2 viability.

Iriarte *et al.* reported that phage viability is clearly affected by exposure to UV irradiation as it causes lesions in DNA, which consequently obstruct RNA transcription and DNA replication [24]. Formulated phage showed better survival after exposure to UV irradiation compared to non-formulated phage. Perhaps γ-PGA can protect the phage from UV irradiation by either masking the phage or due to the turbidity of the solution which refracts the UV radiation. 

The mechanism behind the protective effect of γ-PGA on phage is difficult to elucidate. Phage stained with SYBER green 1 was used to investigate the interaction between the phage and γ-PGA, as the dye is a nucleic-acid stain binding to the genomic DNA of the phage. The resulting complex absorbs blue light and emits green light as shown in (Figure 9). However, as both phage and γ-PGA are negatively charged, electrostatic interactions may not be the mechanism behind their interaction. Divalent cations such as Ca^2+^ and Mg^2+^ may influence the binding kinetics of MS2. Pham *et al.* discovered Ca^2+^ increased attachment efficacies of MS2 to Suwannee River Natural Organic Matter (SRNOM)-coated silica surfaces by up to seventeen times compared to Mg^2+^ [33]. The authors also showed the adsorption of γ-PGA, which was one of the amino-acid residues found on the surface of MS2 capsids present on SRNOM-coated surfaces. This suggests that when γ-PGA and phage are exposed to divalent cations such as Ca^2+^, MS2 has a higher affinity to γ-PGA due to the carboxylic functional groups found on MS2 and γ-PGA surfaces, allowing salt ions to form cation bridges between phage and γ-PGA, as the negative charge density is greater on γ-PGA than on phage [34]. Salt ions could also have a role, by partially neutralizing the negative charges between phage and γ-PGA, increasing the chance of electrostatic interactions. The interaction between γ-PGA and the phage may explain the protective effect of γ-PGA on phage through masking the phage and then may protect it from harsh conditions.

Our work presented in this study with formulated T-even phage and MS2 phage has provided very interesting results, as it is clear that phage stability can be increased by γ-PGA. This may have many implications as γ-PGA could be used to increase the stability of therapeutic bacteriophages, or even other types of virus which are clinically relevant in disease treatment. One major drawback of virus-based vectors is their lack of stability [35], so the use of γ-PGA may widen the range of viruses that could be studied for therapeutic or non-therapeutic benefit. Increasing the range of clinically-relevant viruses could have wide implications for medicine, as viruses can be used for many clinical applications including gene therapy, drug delivery and as chemotherapeutic antibacterial agents. Increasing the stability of viruses could therefore have implications on almost every area of biomedical science.

However, further study into the mechanism of protective effects should focus on the interaction between the phage and the polymer. This could then be used to enhance the association, and therefore the protective effect. Further experimental parameters which are important in environmental survival of viral particles could also be investigated such as humidity and salinity.

## 4. Material and Methods

### 4.1. γ-PGA Production, Purification and Characterization

The biopolymer γ-PGA was produced and isolated as previously described [36]. The obtained polymer was identified by Fourier Transforming Infrared Spectroscopy (FTIR) (Genesis II, Mattson, Geneseo, NY, USA) and Nuclear Magnetic Resonance (^1^H-NMR, Bruker Ultrashield AVANCE II 600 MHz, Rheinstetten, Germany). ^1^H-NMR Spectra were obtained with 64 scans, an 11 ms pulse width, and a 2.65 s acquisition time. The data was collected and analyzed using Bruker TOPSPIN 2.0 software, Deuterium oxide (D_2_O) was used as a solvent. The number average molecular mass (M_n_) was determined by conventional aqueous based gel permeation chromatography (GPC) at Smithers Rapra in Shrewsbury, UK. An MZ Hema guard plus 2× Hema Linear column (Cognis Performance Chemicals Ltd., Southampton, UK) was used for analysis. The GPC experiments were carried out in 0.2 M NaNO_3_, 0.01 M NaH_2_PO_4_, at pH 7, with a flow rate of 1 mL/min at 30 °C. The data was collected and analyzed using Polymer Laboratories “Cirrus 3.0” software.

### 4.2. Bacterial Strains and Phages

Wild type *E. coli* (NCIMB 11291) was used as an host for T4 and T2 bacteriophages, and *E. coli* (NCIMB 9481) was used as an host for MS2 phage. *B. subtilis* natto (ATCC 15245) was used for γ-PGA production. All bacteria strains and phages and were obtained from the National Collection of Industrial and Marine Bacteria (NCIMB, Aberdeen, UK).

### 4.3. Phage Amplification and Titration

Phage was amplified and the titrations were calculated using a standard overlay technique as previously reported [23].

### 4.4. Phage and γ-PGA Formulation

Appropriate amounts of γ-PGA dissolved in Phosphate Buffer Saline (PBS) were autoclaved at 110 °C for 30 min as previously reported [10]. Then 0.5 mL of a phage suspension containing 2.2 × 10^8^ or 10^7^ PFU/mL, was mixed with 4.5 mL of different concentrations of sterilized γ-PGA to obtain final concentration of 0.1, 1 and 2% of γ-PGA and 10^7^ or 10^6^ PFU/mL of phage. Each dilution was then incubated at room temperature for 1 h before use. The term formulated phage means phage with γ-PGA, and non-formulated phage means phage without γ-PGA, just in PBS.

### 4.5. Effect of Elevated Temperature

Formulated and non-formulated phage samples were kept for 3 d in an Eppendorf tube at different temperatures. MS2 phage were kept at 22, 37 and 50 °C and evaluated on each day of exposure while T-even phages were kept at 22, 50, 60 and 70 °C and evaluated after 1, 2, 3, 4 and 24 h using a standard overlay technique as previously described [23], which represent viability, infectivity or survival rate of the phages.

### 4.6. Effect of UV Irradiation on Phage Viability

Initially, 1 mL of formulated and non-formulated phage in 6 wells tissue culture plates were placed into a UV Crosslinker (UVP® 95-0174-01 Model CL-1000, 8-watt, 254 nm Shortwave, 115V Ultra-Violet Products*,* Cambridge, UK). Samples were exposed to UV irradiation for 10 min and evaluated for phage viability after 5 and 10 min using a standard overlay technique as previously described [23].

### 4.7. Effect of Different pH Values on Phage Longevity

The pH of the solutions (0.85% isotonic saline, BR0053 Oxoid Ltd., Basingstoke, UK) was adjusted by adding 0.1 M HCl or 0.1 M NaOH to obtain pH of ~3, 4, 6.4 and 11. Then 0.5 mL of formulated and non-formulated phage samples at 2.2 × 10^7^ PFU/mL, were added to 4.5 mL of each different pH solutions to a final phage concentration of 2.2 × 10^6^ PFU/mL and adjusted again to the desired pH. Samples were tested after 0, 24, 48, and 72 h. However, for pH 3 the samples were tested after 0, 0.5, 1, 2, 3 and 24 h. 

### 4.8. Fluorescence Microscopy with SYBR 1 to Determine γ-PGA-Phage Interaction

Phages were investigated under fluorescence microscopy (Olympus BX61 Fluorescent microscope, Tokyo, Japan) as in a previously described study [37]. 20 μL of formulated and non-formulated T2 were deposited on slides and stained with SYBR green I (ABgene, Epsom, UK). The resulting samples were examined immediately under fluorescence microscopy. Non-formulated T2 was used as a control.

### 4.9. Survival Experiments and Statistical Analyses

All samples from heat, UV and different pH experiments were evaluated using a standard overlay technique [23]. The data ta obtained from different experiments in this study were converted to logarithmic values, the resulting values transformed to survival percentages relative to time zero using MS Excel to give the reader clearer image. Survival levels in formulated and non-formulated phage samples were compared using the Student’s T-test and a one-way analysis of variance (ANOVA) in the statistical package GraphPad Prism version 6.03 (GraphPad Software, Inc., La Jolla, CA, USA). *P* value ≤ 0.05 was considered to be statistically significant.

## 5. Conclusions

The present study suggests that the biodegradable polymer formulation used in this work may be used for better bio-control using phages. The data obtained revealed that γ-PGA does indeed have a protective effect on phage. It was found that γ-PGA protected T even phages from UV damage, damage due to exposure to high temperatures and the exposure to extremes of pH values. The other model phage used in this experiment, MS2, was naturally resistant to varying pH, so the protective effect for pH could not be determined. This result has many wider implications in the field of medicine, and other industries where viruses can be of use, such as agriculture. This is because viruses including bacteriophage can have therapeutic effects. The complexing of γ-PGA with viruses which are being studied in gene therapy may increase the viability of the virus in the human body, increasing the effectiveness of the therapy, could lead to a significant improvement in these treatment modalities.
